# Ultraconserved region-containing *Transformer 2β4* controls senescence of colon cancer cells

**DOI:** 10.1038/oncsis.2016.18

**Published:** 2016-04-04

**Authors:** K Kajita, Y Kuwano, Y Satake, S Kano, K Kurokawa, Y Akaike, K Masuda, K Nishida, K Rokutan

**Affiliations:** 1 Department of Pathophysiology, Institute of Biomedical Sciences, Tokushima University Graduate School, Tokushima, Japan

## Abstract

Ultraconserved regions (UCRs) are >200 bp genomic segments with perfect human-to-rodent sequence identity. Transcribed UCRs constitute a new category of noncoding RNAs whose functions remain poorly understood. The human *transformer 2β* (*TRA2B*) gene contains a 419-bp UCR spanning the 276-bp exon 2 and its neighboring introns. *TRA2B* exon 2 has premature stop codons, whereas an exon 2-containing splice variant (*TRA2β4*) was expressed preferentially in the nuclei of human colon cancer cells. *TRA2β4* knockdown p53-independently stimulated *CDKN1A* transcription and increased p21, resulting in the appearance of senescent cells. Biotin pull-down and RNA immunoprecipitation assays revealed that *TRA2β4* interacted with Sp1 through a Sp1-binding sequence (485-GGGG-488) in a stem-loop structure of exon 2. Mutation of this sequence (485-AAGG-488) disrupted the stem-loop structure, blocked the interaction with Sp1 and increased *CDKN1A* transcription. Overexpression of *TRA2β4* significantly decreased *CDKN1A* mRNA levels and accelerated cell growth, but the introduction of the mutation in the Sp1-binding sequence completely canceled these effects. Taken together, *TRA2β4* may sequester Sp1 from occupying promoters of target genes including *CDKN1A*, promoting cell growth by interrupting the senescence-related gene expression program. This novel function of *TRA2β4* may uncover an oncogenic function of transcribed UCRs.

## Introduction

Alternative splicing of pre-mRNAs generates protein diversity from a limited number of genes.^1^ The utilization of alternative splice sites is regulated by *cis*-acting elements and *trans*-acting factors through the interaction of a family of serine/arginine (SR)-rich splicing factors (SRSFs) and heterogeneous nuclear ribonucleoproteins. Alternative splicing occurs in a developmental stage-, sex- or tissue-specific manner and in response to the surrounding microenvironment.^2, 3^ High-throughput RNA sequencing of tissue-specific splicing events indicates that >90% of human genes produce multiple spliced isoforms.^3^ At the same time, aberrant alternative splicing participates in many genetic and acquired diseases including cancer.^2, 4^

**Figure 1:**
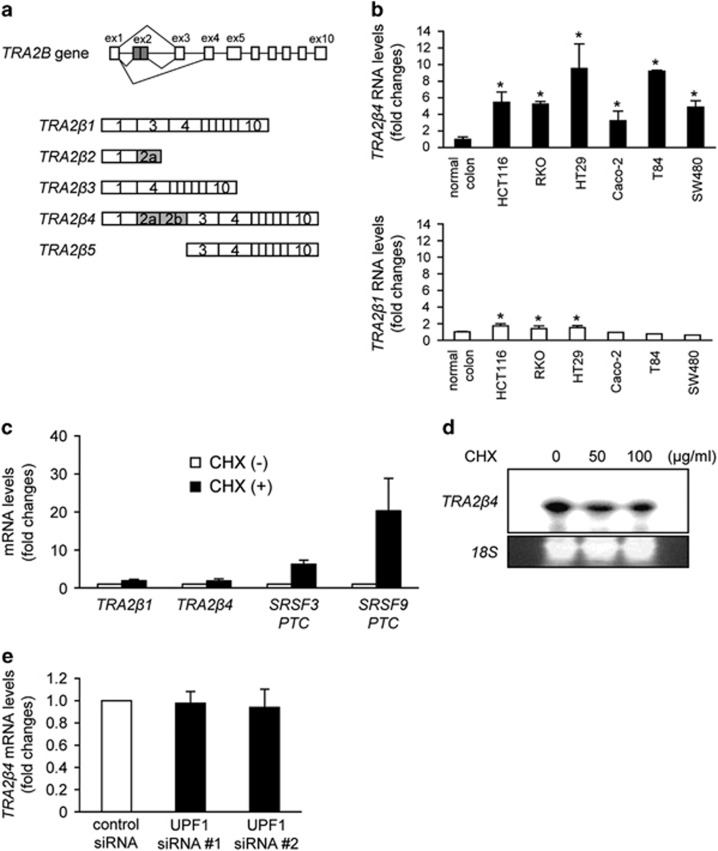
Expression of *TRA2β1* and *TRA2β4* in colon cancer cell lines. (**a**) Schematic diagram of the human *TRA2B* gene. Exons (ex) are indicated by open boxes and Arabic numbers. Filled boxes denote the ultraconserved exon 2. Five splice variants generated from *TRA2B* and the use of each exon are shown. (**b**) Amounts of *TRA2β4* (upper panel) and *TRA2β1* (lower panel) mRNAs in normal human colon and colon cancer cell lines (HCT116, RKO, HT29, Caco-2, T84 and SW480) were measured by qPCR using *GAPDH* mRNA as an endogenous quantity control. Values are means±s.d. from three independent experiments. *Significantly different by analysis of variance (ANOVA) and Bonferroni test (*P*<0.05). (**c**) HCT116 cells were treated with 10 μg/ml cycloheximide (CHX) for 4 h to inhibit NMD. Then, changes in mRNA levels of *TRA2β1, TRA2β4*, *SRSF3 PTC* and *SRSF9 PTC* were measured by qPCR using *GAPDH* mRNA as an endogenous quantity control. Values are means±s.d. from three independent experiments. (**d**) After treatment of HCT116 cells with 50 or 100 μg/ml CHX for 4 h, *TRA2β4* levels were assayed by Northern hybridization with a locked nucleic acid (LNA) probe targeting for exon 2*. 18S* rRNA was used as a loading control. (**e**) After treatment of HCT116 cells with 10 nm of two different *UPF1* siRNAs for 48 h, changes in *TRA2β4* mRNA levels were measured by qPCR using *GAPDH* mRNA as an endogenous quantity control. Values are means±s.d. from three independent experiments.

An SR-like protein transformer 2β (Tra2β) regulates splice site selection of several genes encoding calcitonin/calcitonin gene-related peptide (*CGRP*), survival motor neuron 1 (*SMN1*) and microtubule-associated protein tau (*TAU*) in a concentration-dependent manner.^5, 6, 7^ Several lines of evidence suggest that overexpressed Tra2β may be involved in the pathogenesis of cancer.^8, 9, 10^ The human *TRA2β* (*TRA2B*) gene is composed of 10 exons and 9 introns. *TRA2B* contains a 419-bp genomic segment with perfect human-to-rodent sequence identity, termed the ultraconserved region (UCR; uc.138, see Supplementary Figure S1) spanning the 276-bp exon 2 and its neighboring introns.^11, 12^ *TRA2B* generates five mRNA isoforms (*TRA2β1–5*) through alternative splicing and usage of alternative promoters or polyadenylation sites (Figure 1a).^12^ Because of the existence of multiple premature termination codons (PTCs) in *TRA2B* exon 2, an exon 2-containing *TRA2β4* mRNA isoform (hereafter referred as *TRA2β4*) is not translated and should be actively degraded through nonsense-mediated mRNA decay (NMD), a surveillance mechanism that degrades PTC-containing mRNAs. However, oxidative stress specifically facilitated *TRA2β4* isoform production in rat gastric mucosa and a human gastric cancer cell line (AGS).^13^

Recently, it has become apparent that non-protein-coding RNAs (ncRNAs) are functionally important for normal development and physiology, as well as for pathologic processes.^14^ ncRNAs can be broadly classified into small (18–200 nt) and long ncRNAs (200 nt to >100 kb).^15^ Among them, epigenetic and genetic defects in a class of small ncRNAs called microRNAs are now recognized as a common hallmark of cancer. However, other ncRNAs, such as transcribed UCRs (T-UCRs), small nucleolar RNAs, PIWI-interacting RNAs or large intergenic noncoding RNAs, may also contribute to the development of many different diseases. There are 481 described UCRs, and more than half of them do not encode any protein.^11^ However, 68% of UCRs are transcribed, constituting a new category of ncRNAs, the T-UCRs.^16^ The wide distribution in the genome and lack of natural variation in the human population suggest an essential functional role in normal cells.^11, 17^ Recent genome-wide expression profiling studies have shown that certain T-UCRs are differentially expressed in human leukemias and carcinomas.^16, 18, 19^ Expression of those UCRs was suggested to be regulated by microRNAs, and the inhibition of an overexpressed T-UCR (uc. 73) induced apoptosis of colon cancer cells.^16^ T-UCRs are widely expressed in neuroblastomas and their expression correlates with important clinicogenetic parameters such as *MYCN* amplification status.^20^ However, the functional role of T-UCRs in cancer development is largely unknown.

Here, we introduce *TRA2β4* as a functional T-UCR preferentially expressed in colon cancer cells. *TRA2β4* may exert an oncogenic function by controlling senescence of colon cancer cells.

## Results

### Colon cancer cells upregulate *TRA2β4* mRNA isoform expression

PTC-containing *TRA2β4* mRNA isoform (*TRA2β4*) was considered to be degraded through NMD. However, when expression levels of *TRA2β4* and *TRA2β1* were examined in different colon cancer cell lines, all cell lines tested expressed significant amounts of *TRA2β4* (Figure 1b, upper panel). *TRA2β4* levels were estimated to be 10–20% of those of *TRA2β1* mRNA that encodes a full-length Tra2β protein. On the other hand, *TRA2β1* mRNA was not consistently overexpressed in the colon cancer cell lines (Figure 1b, lower panel). Human colon cancer (HCT116) cells expressed mainly *TRA2β1* mRNA and increased *TRA2β4* isoform production after exposure to sodium arsenite (Supplementary Figures S2b and c) as similarly observed in gastric cancer cells.^13^

Inactivation of NMD with cycloheximide increased the amounts of PTC-containing mRNA variants of *SRSF3* and *SRSF9* (Figure 1c). In contrast, cycloheximide treatment failed to increase *TRA2β4* levels, which was confirmed by quantitative real-time reverse transcription– (qPCR) (Figure 1c) and Northern blot analysis (Figure 1d). Two different small interfering RNAs (siRNAs) targeting a key regulator of NMD (UPF1) effectively reduced *UPF1* mRNA levels (Supplementary Figure S3a) and significantly increased the amounts of *SRSF3* and *SRSF9* PTC variants (Supplementary Figure S3b), but they did not increase *TRA2β4* (Figure 1e).

**Figure 2:**
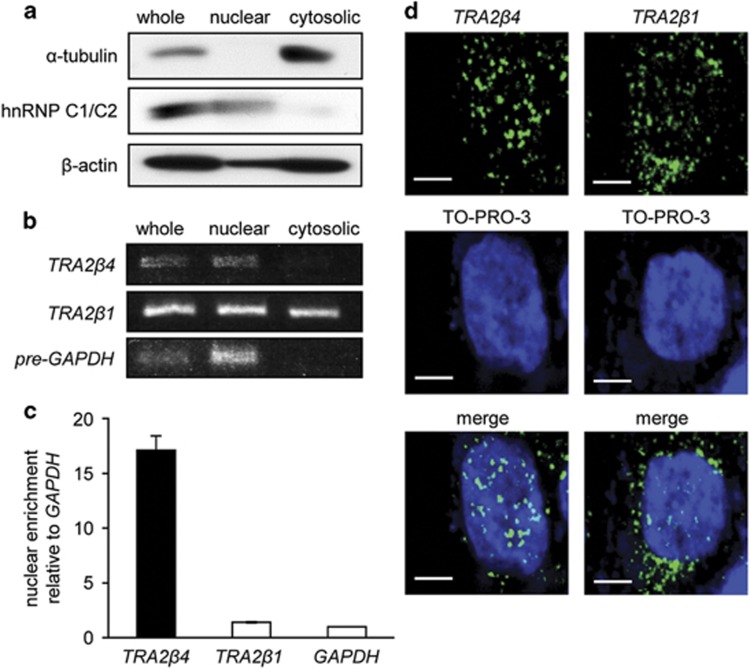
Subcellular distribution of *TRA2β4* in HCT116 cells. (**a**) After nuclear and cytosolic fractions were prepared from HCT116 cells, the purity of each fraction was monitored by measuring a cytosolic protein (α-tubulin) and a nuclear protein (heterogeneous nuclear ribonucleoprotein (hnRNP) C1/C2) by western blotting. (**b**) Total RNA was extracted from each fraction and *TRA2β1* mRNA or *TRA2β4* was amplified by RT–PCR using specific primer sets. The purity of each faction was also assessed by RT–PCR measurement of *GAPDH* pre-mRNA. (**c**) *TRA2β1*, *TRA2β4* and *GAPDH* mRNA levels in each fraction were measured by qPCR. Nuclear-to-cytosolic distribution ratio of each mRNA was calculated. Nuclear enrichment of *TRA2β1* mRNA or *TRA2β4* is indicated by comparing values to nuclear/cytosolic distribution ratio of *GAPDH* mRNA. Values are means±s.d. from three independent experiments. (**d**) Subcellular localization of *TRA2β4* and *TRA2β1* mRNA in HCT116 cells was examined by RNA fluorescence *in situ* hybridization (RNA-FISH) using locked nucleic acid (LNA) probes against exon 2 and exon 1–3 junction of *TRA2β1* mRNA (green), respectively, as described in Materials and methods section. Cells were counterstained with TO-PRO-3 (blue). Scale bars, 5 μm.

To explain why NMD did not control *TRA2β4* expression, we prepared nuclear and cytoplasmic fractions from HCT116 cells. The purity of each fraction was confirmed by western blotting using antibodies for cytosolic (α-tubulin) and nuclear (heterogeneous nuclear ribonucleoprotein C1/C2) marker proteins (Figure 2a), and by reverse transcription–PCR (RT–PCR) using primers targeting *glyceraldehyde-3-phosphate dehydrogenase* (*GAPDH*) pre-mRNA (Figure 2b). RT–PCR amplified *TRA2β4* in nuclear fractions containing *GAPDH* pre-mRNA, but not in cytosolic fractions, whereas *TRA2β1* mRNA was distributed in both nuclear and cytoplasmic fractions (Figure 2b). qPCR measurement showed that *TRA2β4* was significantly enriched in the nucleus, compared with *TRA2β1* and *GAPDH* mRNAs (Figure 2c). RNA fluorescence *in situ* hybridization revealed that overexpressed *TRA2β4* signals were present preferentially in the nuclei, whereas *TRA2β1* mRNA signals were distributed in both the nucleus and the cytoplasm (Figure 2d). We also confirmed that HeLa cells constitutively expressed *TRA2β4* in their nuclei similar to a nucleus-retained noncoding RNA (*MEN ɛ/β*) (Supplementary Figure S4a). Transiently overexpressed *TRA2β4* was retained predominantly in the nuclei of HEK293T as well as of HeLa cells (Supplementary Figure S4b). These results suggest that significant amounts of *TRA2β4* are retained in the nucleus and may escape from NMD-mediated degradation.

### *TRA2β4* knockdown inhibits cell growth

**Figure 3:**
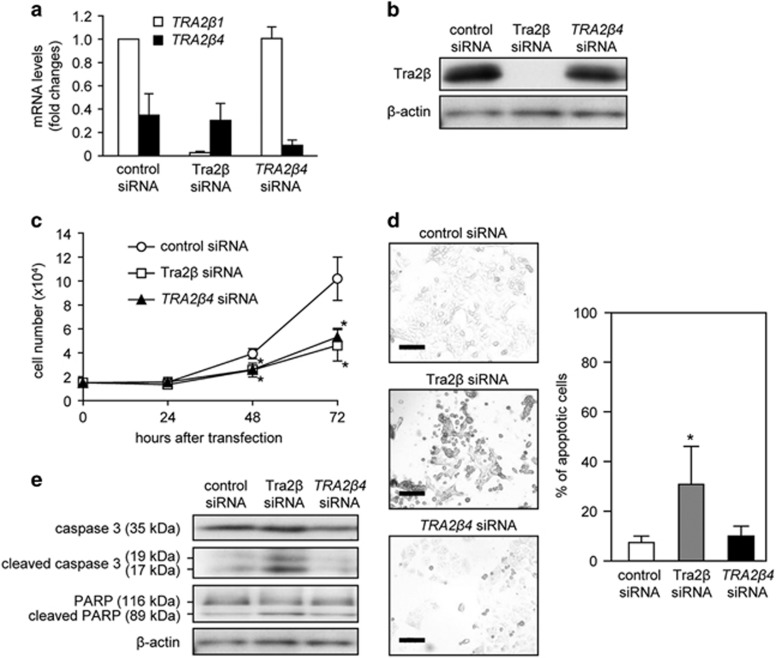
Effect of *TRA2β4* silencing on cell proliferation and apoptosis. (**a**) After HCT116 cells were treated with 10 nm of *TRA2β4* or Tra2β siRNA for 48 h, *TRA2β1* mRNA and *TRA2β4* levels were measured by qPCR using *GAPDH* as an endogenous quantity control. Values are means±s.d. from six independent experiments. (**b**) HCT116 cells were treated with 10 nm *TRA2β4*, Tra2β or control siRNA for 48 h, and then the amounts of Tra2β were measured by western blotting using β-actin as a loading control. (**c**) HCT116 cells (1.5 × 10^4^ cells) were seeded in 35-mm-diameter dishes and transfected with 10 nm *TRA2β4*, Tra2β or control siRNA. Subsequently, growing cells were harvested and counted at the indicated times. Values are means±s.d. from four independent experiments. *Significantly different by analysis of variance (ANOVA) and Bonferroni test (*P*<0.05). (**d**) After HCT116 cells were treated with 10 nm *TRA2β4*, Tra2β or control siRNA for 24 h, they were labeled using the DeadEnd Colorimetric TUNEL system (left panels), and the percentages of TUNEL-positive cells were determined (right panel). Values are means±s.d. from three independent experiments. *Significantly different by ANOVA and Bonferroni test (*P*<0.05). Scale bars, 50 μm. (**e**) After treatment of HCT116 cells with 10 nm *TRA2β4*, Tra2β or control siRNA for 48 h, whole-cell lysates were prepared from these cells. The levels of unprocessed or cleaved caspase-3 and PARP were measured by western blotting using β-actin as a loading control.

To test the possibility that the *TRA2β4* variant might be a functional T-UCR, we prepared two different siRNAs against *TRA2β* mRNAs (Tra2β siRNA targeting exon 6 and *TRA2β4* siRNA targeting exon 2) and examined the effects of these siRNAs on cell growth. Although Tra2β siRNA was designed to degrade both *TRA2β1* and *TRA2β4* isoforms, it degraded *TRA2β1* more effectively than *TRA2β4* probably because of the predominant expression of the *TRA2β1* isoform (Figure 3a). Consequently, Tra2β siRNA effectively reduced Tra2β protein (Figure 3b). In contrast, *TRA2β4* siRNA selectively reduced *TRA2β4* without changing *TRA2β1* mRNA and Tra2β protein levels (Figures 3a and b). Treatment with Tra2β siRNA significantly inhibited cell growth (Figure 3c) in association with an increase in terminal deoxynucleotidyl transferase-mediated UTP end labeling (TUNEL)-positive cells (Figure 3d) and activation of poly(ADP-ribose) polymerase (PARP) and caspase-3 (Figure 3e). It was of interest that *TRA2β4* siRNA did not change *TRA2β1* mRNA or Tra2β protein levels, but it did inhibit cell growth (Figure 3c). Moreover, the selective *TRA2β4* knockdown did not significantly increase TUNEL-positive cells (Figure 3d) and did not activate PARP and caspase-3 (Figure 3e).

### *TRA2β4* knockdown induces cellular senescence

**Figure 4:**
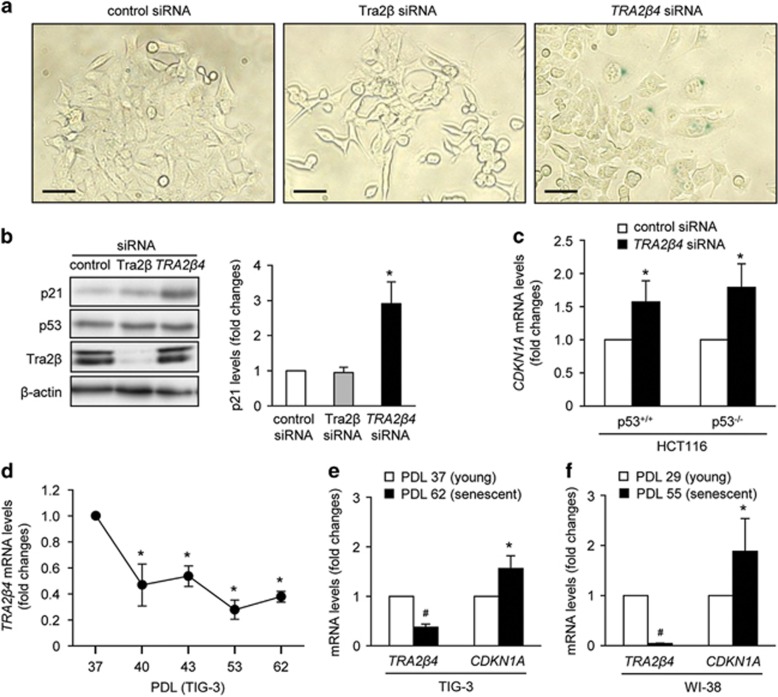
*TRA2β4* knockdown induces cellular senescence. (**a**) HCT116 cells were transfected with 10 nm Tra2β, *TRA2β4* or control siRNA for 72 h. They were then subjected to SA-β-gal staining. Scale bars, 10 μm. (**b**) After the transfection for 48 h, amounts of p21and p53 were measured by western blotting with respective antibodies (left panel). P21 levels were quantified using the Image J software (NIH, Bethesda, MD, USA) (right panel). β-Actin was used as a loading control. (**c**) After wild-type (p53^+/+^) or p53^−/−^ HCT116 cells were treated with 10 nm *TRA2β4* or control siRNAs for 48 h, *CDKN1A* mRNA levels were determined by qPCR using *GAPDH* mRNA as an endogenous quantity control. Data are expressed as fold changes compared with those in control siRNA-treated cells. Values are means±s.d. (*n*=5). (**d**) *TRA2β4* levels in TIG cells at population doubling levels (PDL) 37, 40, 43, 53 and 62 were measured by qPCR using *GAPDH* mRNA as an endogenous quantity control. Data were expressed as fold changes relative to the levels in PDL 37 TIG-3 cells. Values are means±s.d. (*n*=3). (**e** and **f**) *TRA2β4* and *CDKN1A* mRNA levels in young (PDL 37) and senescent (PDL 62) TIG-3 cells as well as young (PDL 29) and senescent (PDL 55) WI-38 cells were measured by qPCR using *GAPDH* mRNA as an endogenous quantity control. Values are means±s.d. from three independent experiments. *Significantly different by analysis of variance (ANOVA) and Bonferroni test (*P*<0.05) compared with those in control siRNA-treated cells.

As shown in Figure 4a, *TRA2β4* siRNA-treated HCT116 cells displayed unique morphological changes, that is, the cells spread out their cytoplasm and became thinner. These enlarged cells were positive for senescence-associated β-galactosidase (SA-β-gal) (Figure 4a). To confirm that *TRA2β4* siRNA induced cellular senescence, we examined the expression of cyclin-dependent kinase inhibitors that have a crucial role in cell-cycle arrest and induction of senescence.^21^ Owing to a frameshift mutation in the *INK4A* gene and the hypermethylated promoter, p16 protein was undetectable in HCT116 cells.^22^ As shown in Figure 4b, treatment with *TRA2β4* siRNA, but not with Tra2β1 siRNA, increased p21 levels. To avoid off-targeting effects, an additional siRNA targeting another sequence in *TRA2β4* (*TRA2β4* siRNA no. 2) were used. Transfection of *TRA2β4* siRNA no. 2 also increased *CDKN1A* mRNA and p21 protein levels, and the percentage of SA-β-gal-positive cells (Supplementary Figure S5). It should be noted that *TRA2β4* knockdown did not induce p53 (Figure 4b), and that *TRA2β4* siRNA-mediated p21 induction occurred even in p53-null (p53^−/−^) HCT116 cells (Figure 4c). These results suggested that the reduction of *TRA2β4* might increase *CDKN1A* mRNA and p21 protein levels in a p53-independent manner. We also confirmed that *TRA2β4* knockdown similarly induced morphological changes and SA-β-gal in p53^−/−^ HCT116 cells (Supplementary Figures S6a and b).

A human fibroblast cell line (TIG-3) exhibited an age-associated reduction of *TRA2β4* levels (Figure 4d). Reciprocal changes in *TRA2β4* and *CDKN1A* mRNA expression were also observed along with senescence of TIG-3 and another human fibroblast cell line (WI-38); senescent cells decreased *TRA2β4* expression in association with increased expression of *CDKN1A* mRNA (Figures 4e and f). These results suggest that *TRA2β4* may regulate p21 expression during replicative senescence.

### TRA2β4 interacts with Sp1 and regulates *CDKN1A* transcription

**Figure 5:**
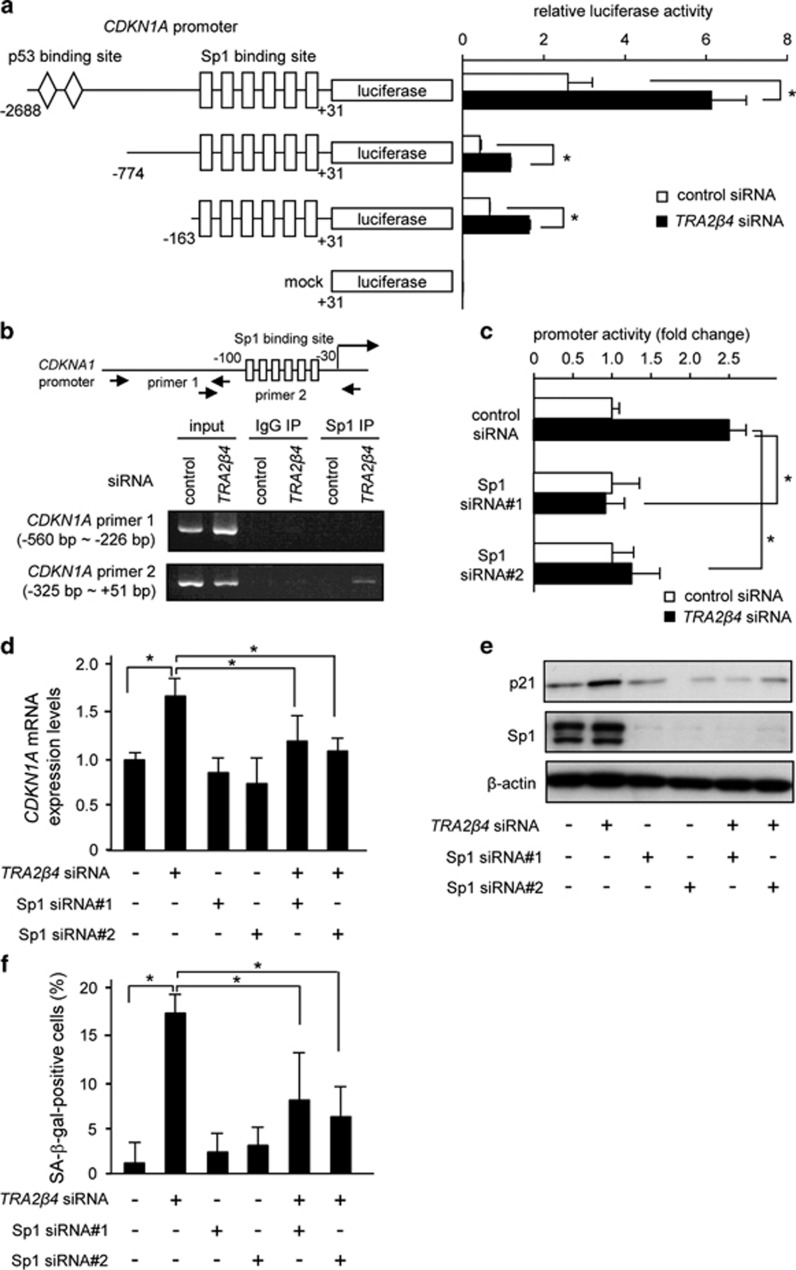
*TRA2β4* modifies promoter activity of the *CDKN1A* gene. (**a**) Twenty-four hours after transfection with 10 nm *TRA2β4* or control siRNA, HCT116 cells were transiently transfected with luciferase reporter plasmids driven by −2688/+31, −774/+31 or −163/+31 bp promoter fragments of *CDKN1A* for 24 h. Luciferase activities in these cells were measured using the Dual-Luciferase Reporter Assay System. *Significantly decreased compared with control siRNA-treated cells (*P*<0.05 by analysis of variance (ANOVA) and Bonferroni test). (**b**) After treatment with *TRA2β4* or control siRNA for 48 h, HCT116 cells were subjected to chromatin immunoprecipitation (ChIP) assays. Formaldehyde-crosslinked nuclear extracts were immunoprecipitated with an anti-Sp1 antibody or normal rabbit IgG (IgG). PCR was performed using an input nuclear chromatin fraction as a template (input). Specific PCR products corresponding to the region of the *CDKN1A* promoter containing the Sp1-binding sites were amplified and separated by agarose gel electrophoresis followed by ethidium bromide staining. (**c**) After treatment with 10 nm Sp1, *TRA2β4* or control siRNA for 24 h, HCT116 cells were transiently transfected with the luciferase plasmid (pGL3-*CDKN1A* −163/+31) for 24 h. Luciferase activities in these cells were measured using the Dual-Luciferase Reporter Assay System. Values are means±s.d. (*n*=4). *Significantly different (*P*<0.05 by ANOVA and Bonferroni test). (**d** and **e**) After HCT116 cells were treated with *TRA2β4* and/or Sp1 siRNA nos 1/2 as indicated for 24 h, expression levels of *CDKN1A* mRNA and p21 were analyzed by qPCR and western blotting. (**f**) After silencing of *TRA2β4* and/or Sp1 nos 1/2, the cells were stained with SA-β-gal and then one hundred cells per individual sample in three independent fields were measured. *Significantly different (*P*<0.05 by ANOVA and Bonferroni test).

*TRA2β4* knockdown did not affect the stability of *CDKN1A* mRNA (Supplementary Figure S7a) and p21 protein (Supplementary Figure S7b) or the translation rate of p21 (Supplementary Figure S7c), suggesting that the reduction of *TRA2β4* may facilitate *CDKN1A* transcription. To confirm this, we cloned the 5′ flank of *CDKN1A* (from −2688 to +31 bp), and HCT116 cells were transfected with a luciferase reporter construct containing −2688/+31, −774/+31 or −163/+31 bp region of the human *CDKN1A* promoter (Figure 5a). The −2688/+31 bp region contained two p53-binding sites and exhibited basal and *TRA2β4* siRNA-mediated upregulation of *CDKN1A* promoter activity (Figure 5a). The −163/+31 bp region as well as the −774/+31 bp region lost the p53-binding sites and markedly reduced the basal promoter activity. However, these two regions still responded to *TRA2β4* knockdown and increased the luciferase activity. The −163/+31 bp region contained a cluster of Sp1-binding sites that likely mediated the response to *TRA2β4* knockdown. To further investigate how reduction of *TRA2β4* resulted in the stimulation of *CDKN1A* transcription, we performed chromatin immunoprecipitation assays with an anti-Sp1 antibody using control or *TRA2β4* siRNA-treated HCT116. Sp1 did not bind to the -560/-226 region or the −325/+51 bp region in control siRNA-treated cells (Figure 5b), whereas in *TRA2β4* siRNA-treated cells, Sp1 bound to the -325/+51 bp region containing the cluster of Sp1-binding sites, but not to the −560/−226 bp region (Figure 5b). These results suggest that Sp1 binding to the *CDKN1A* promoter may be blocked when significant amounts of *TRA2β4* are present in the nucleus of HCT116 cells. The crucial role of Sp1 was also confirmed by two additional findings. That is, knockdown of Sp1 almost significantly blocked the *TRA2β4* silencing-induced upregulation of *CDKN1A* promoter activity (Figure 5c), *CDKNA1A* mRNA expression (Figure 5d), and p21 protein expression (Figure 5e). In addition, co-transfection of Sp1 siRNA with *TRA2β4* siRNA reduced *TRA2β4*-mediated increase in the percentage of SA-β-gal-positive cells (Figure 5d). Thus, the Sp1 elements seemed to play a pivotal role in the transcriptional activation of *CDKN1A* after silencing of *TRA2β4*.

**Figure 6:**
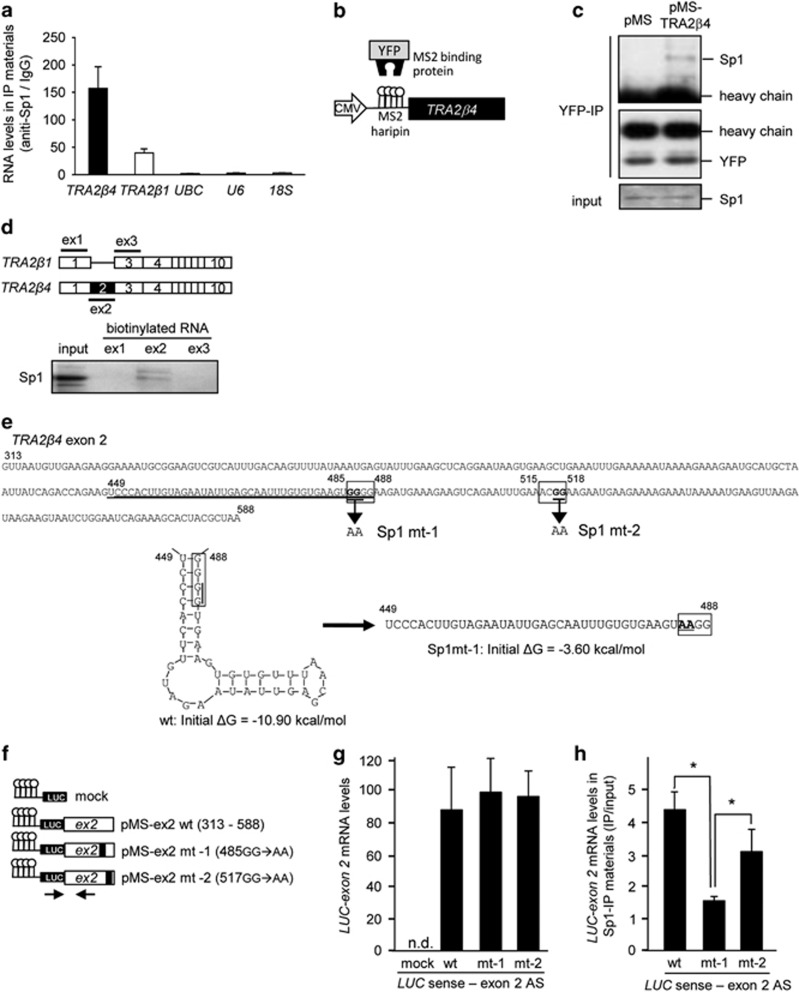
*TRA2β4* binds to Sp1 via exon 2. (**a**) Nuclear lysates prepared from UV-crosslinked HCT116 cells were subjected to an RIP assay using an anti-Sp1 antibody or normal mouse IgG. Immunoprecipitated RNAs were quantified by qPCR. Data are shown as enrichment relative to values obtained with normal mouse IgG. Values are means±s.d. (*n*=4). (**b**) Scheme for full-length *TRA2β4* bearing bacteriophage MS2 hairpins (pMS-*TRA2β4).* YFP, yellow fluorescence protein. (**c**) After HCT116 cells were co-transfected with pMS2-YFP and pMS-*TRA2β4* or control mock (pMS), association between Sp1 and *TRA2β4* was analyzed using immunoprecipitaion with anti-YFP antibody and western blotting with anti-Sp1 antibody. (**d**) Nuclear fractions (40 μg) prepared from HCT116 cells were incubated with 1 μg of biotinylated transcripts designed as exon 1 (ex1), exon 2 (ex2) and exon 3 (ex3) of *TRA2β4* in 10 mm Tris-HCl buffer, pH 8.0, containing 1 mm EDTA, 250 mm NaCl and 0.5% Triton X-100 for 1 h at room temperature. RNA–protein complexes were isolated with paramagnetic streptavidin-conjugated Dynabeads, and bound Sp1 was detected by western blotting. (**e**) Nucleotide sequence of *TRA2β4* exon 2. Two consensus Sp1-binding sites are boxed. Formation of stem-loop structure of *TRA2β4* exon 2 RNA (449–488 nt) and its disruption by the mutation of 485-GGGG-488 to 485-AAGG-488 are shown below. These structures were predicted using CentroidFold and M-FOLD programs. (**f**) Scheme for pMS2-LUC (*Renilla* luciferase gene (*LUC*)), pMS2-LUC-exon 2 wild-type (pMS-ex2 wt), pMS2-LUC-exon 2 mt-1 (pMS-ex2mt-1), and pMS2-LUC-exon 2 mt-2 (pMS-ex2mt-2). Arrows indicate a primer set used. (**g** and **h**) Nuclear lysates prepared from UV-crosslinked HCT116 cells were subjected to an RIP assay using an anti-Sp1 antibody. Immunoprecipitated *LUC*-fused exon 2 RNAs were measured by qPCR. Data are shown as enrichment relative to values obtained from amount of each input. Values are means±s.d. (*n*=4).

As shown in Figure 6a, RNA immunoprecipitation (RIP) assays indicated that an anti-Sp1 antibody retrieved endogenous *TRA2β4* more effectively than *TRA2β1* mRNA or other control RNAs, such as *UBC*, *U6* and *18S*, suggesting the association between Sp1 and *TRA2β4*. We prepared a plasmid encoding full-length *TRA2β4* bearing bacteriophage MS2 hairpins (pMS-*TRA2β4*; Figure 6b). HCT116 cells were co-transfected with pMS-*TRA2β4* and pMS2-yellow fluorescence protein (YFP) bearing an YFP-fused MS2-binding protein.^23^ Using this system, we again confirmed the association between MS2-tagged *TRA2β4* and Sp1 in YFP-immunoprecipitated materials (Figure 6c). The presence of exon 2 in *TRA2β4* is the only difference between *TRA2β4* and *TRA2β1*, biotin-pull-down assays with HCT116 cells transcribing *TRA2β* exon 1, 2 or 3 RNA demonstrated the specific association of Sp1 with *TRA2β* exon 2 RNA (Figure 6d). As shown in Figure 6e, *TRA2β4* exon 2 contains two Sp1 consensus sequences (485-GGGG-488 and 515-ACGG-518). CentroidFold (http://www.ncrna.org/centroidfold) and M-FOLD (http://mfold.rna.albany.edu/?q=mfold) programs indicate that *TRA2β4* contains a stem-loop structure within exon 2 (449–488 nt, underlined in Figure 6e). Both programs also indicate that the introduction of two-point mutations at the Sp1-binding sequence (exon 2 mt-1, 485-GGGG-488 to 485-AAGG-488) completely disrupts the stem-loop structure (Figure 6e). The *MS2* RNA hairpin-tagging system was again used to identify the interaction between exon 2 and Sp1. We constructed pMS2-LUC (*Renilla* luciferase gene (*LUC*))-exon 2 wild-type (exon 2 wt), pMS2-LUC-exon 2 mt-1 and pMS2-LUC-exon 2 mutated at another Sp1-binding sequence (exon 2 mt-2, 515-ACGG-518 to 515-ACAA-518) (Figure 6f) and transfected into HCT116 cells (Figure 6g). RIP between Sp1 and *LUC*-fused exon 2 constructs indicated that two-point mutations in the stem-loop motif (exon 2 mt-1), but not in another Sp1-binding site (exon 2 mt-2), significantly blocked the association between *TRA2β4* exon 2 and Sp1 (Figure 6h), suggesting a crucial role of the consensus Sp1-binding site (485-GGGG-488) within the stem-loop motif.

We also examined whether the interaction between *TRA2β4* and Sp1 was actually involved in regulating the expression of other Sp1-regulated genes, and found that *TRA2β4* knockdown significantly increased the expression of *KLF5*, *DRG2* and *PRKRA* mRNAs (Supplementary Figure S8). Sp1 is crucial for transcription of these genes.^24, 25, 26^

### Expression of *TRA2β4* in colon cancer cells promotes cell growth

**Figure 7:**
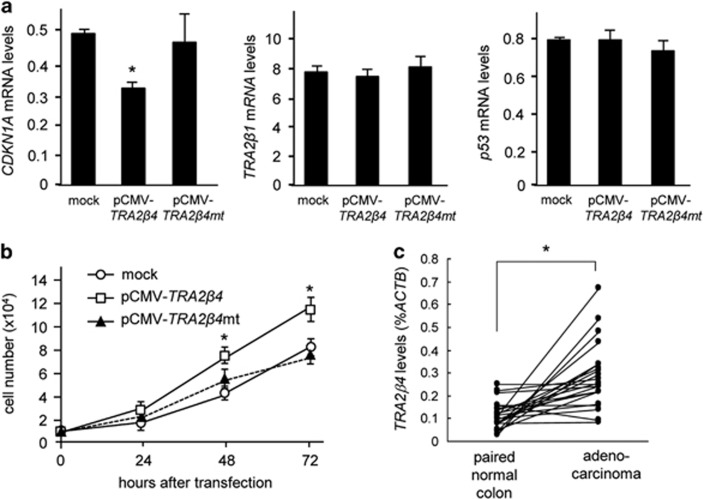
Expression of *TRA2β4* in colon cancers. (**a**) HCT116 cells were transfected with pcDNA3.1 (mock), pCMV-*TRA2β4* or pCMV-*TRA2β4* mutated in the stem-loop motif (pCMV-*TRA2β4mt*, 485-AAGG-488) for 48 h. mRNA levels were measured by qPCR using *GAPDH* as an endogenous quantity control. *Significantly decreased compared with mock-treated cells (*P*<0.05 by Student's *t*-test). (**b**) After transfection of HCT116 cells (1 × 10^4^ cells per 24-well dish) with pcDNA3.1 (mock), pCMV-*TRA2β4* or pCMV-*TRA2β4mt*, the number of growing cells were measured using CellTiter96 AQueous Cell Proliferation Assay (MTS). Values expressed as means±s.d. (*n*=3). *Significantly increased compared with mock-transfected cells (*P*<0.05 by Student's *t*-test). (**c**) Using human colon cancer tissue qPCR arrays (TissueScan, HCRT103), *TRA2β4* expressed in cDNAs from adenocarcinomas of the colon and surrounding normal colon tissues were measured by qPCR in 24 patients. Values were normalized to *ACTB* mRNA levels.

To investigate the possible roles of *TRA2β4* in human colon cancer, we generated a pCMV construct encoding *TRA2β4* (pCMV-*TRA2β4*) or *TRA2β4* containing the mutation (exon 2 mt-1, 485-GGGG-488 to 485-AAGG-488) in the stem-loop motif (pCMV-*TRA2β4mt*). Overexpression of *TRA2β4* could significantly decease *CDKN1A* mRNA levels and accelerate cell growth, but the introduction of the mutation completely cancel these effects (Figures 7a and b). We also confirmed that the increased levels of *TRA2β4* or *TRA2β4mt* did not affect *TRA2β1* and *p53* mRNA levels (Figure 7a).

Finally, we measured the expression of *TRA2β4* in 24 different cDNA libraries prepared from patients with colon cancer (Figure 7c). The relative expression of *TRA2β4* varied depending on individual paired samples; however, colon cancer tissues expressed significantly higher levels of *TRA2β4*, compared with surrounding normal tissues.

## Discussion

Among 481 UCRs discovered, 325 UCRs are transcribed and implicated to function as noncoding RNAs, whereas their specific functions are not fully investigated.^16^ We show here that human colon cancer cells express significant amounts of a T-UCR (*TRA2β4*), which is transcribed from uc 281. It has been shown that aberrant expression of specific T-UCRs is associated with chronic lymphocytic leukemia, colorectal cancer and hepatocellular carcinoma.^16, 18, 19^ We previously reported that Hu antigen R regulated alternative splicing of *TRA2β* to selectively produce *TRA2β4* in human colon cancer cells under oxidative stress.^27^ Although *TRA2β4* is a PTC variant, transcribed *TRA2β4* was retained preferentially within the nucleus and resistant to the RNA surveillance NMD. Distinct ncRNAs retained within the mammalian cell nucleus are now referred to as nuclear-retained regulatory RNAs, and they are suggested to have structural roles or act as riboregulators.^15, 28, 29^ We suggest here that *TRA2β4* may function as a novel regulator of senescence in colon cancer cells.

Tra2β is overexpressed in several types of cancers and has been suggested to participate in their abnormal growth.^2, 8^ In fact, our previous studies showed that Tra2β knockdown inhibited proliferation of colon cancer cells and induced their apoptotic cell death.^9, 10^ In the present study, we found that *TRA2β4* (rather than Tra2β-encoding *TRA2β1*) was consistently overexpressed in all colon cancer cell lines tested. Although selective reduction of *TRA2β4* inhibited cell growth, it did not stimulate apoptosis, but facilitated cellular senescence. These results suggest novel functions of *TRA2β4* distinct from those of Tra2β protein. Cellular senescence is regulated by multiple factors, including p53, pRb and cyclin-dependent kinase inhibitors (p21, p16^INK4a^, p14^ARF^ and p15^INK4b^). p16^INK4a^ is one of the crucial factors for senescence in human tumors.^30^ However, like many other cancer cell lines, the *p16INK4A* gene is silenced in HCT116 cells owing to a frameshift mutation and the hypermethylated promoter.^22^ In contrast, *TRA2β4* knockdown, but not Tra2β knockdown, induced p21 without changing p53 expression. The induction of p21 was observed even in p53^−/−^ HCT116 cells. Upregulation of p21 induces senescence and the inactivation of this protein prevents senescence of colon cancer cells.^31, 32^ Thus, p21 was likely to be one of the key factors in the *TRA2β4* knockdown-induced growth arrest and senescence of HCT116. Replicative senescence-dependent decline of *TRA2β4* levels and a reciprocal induction of p21 observed in human fibroblast cell lines also support an important role of *TRA2β4* in the regulation of cellular senescence. *TRA2β4* knockdown could increase p21 levels in a p53-independent manner and it did not change either the stability or translation rate of *CDKN1A* mRNA. Based on the results, we speculated that *TRA2β4* might directly regulate transcription of the *CDKN1A* gene, and found that *TRA2β4* interacted with Sp1 via exon 2 and regulated *CDKN1A* mRNA expression. These results suggest that aberrantly expressed *TRA2β4* may prevent Sp1 from occupying promoters of target genes, including *CDKN1A*, and thus promote cell survival by interrupting the senescence-related gene expression program. Sp1 is crucial for basal transcription of *KLF5*, *DRG2* and *PRKRA*.^24, 25, 26^ We confirmed that *TRA2β4* knockdown significantly increased the expression of these mRNAs in HCT116 cells, suggesting that interactions between *TRA2β4* and Sp1 may be able to significantly alter the gene expression program.

Although the precise mechanism underlying the function of ncRNAs remains poorly understood, one emerging theme is the interaction between ncRNAs and protein complexes.^33, 34^ Several ncRNAs are required for the precise localization of chromatin proteins on genomic DNA targets.^35^ ncRNAs can also regulate the activity of protein complexes. An ncRNA upstream of *CCND1* (ncRNA_*CCND1*_) and the *NRON* ncRNA can bind to RNA-binding proteins or transcription factors and change their activities.^36, 37^ Distinct ncRNAs can work as molecular ‘decoys'. For example, the *GAS5* ncRNA binds to the glucocorticoid receptor, blocking the correct binding to its regulatory elements.^38^ The long ncRNA *PANDA* interacts with the transcription factor NF-YA and limits the expression of proapoptotic genes, leading to apoptosis.^39^ At present, however, any T-UCR–protein interaction has not been documented.

*TRA2β* exon 2 has four purine-rich exonic splicing enhancers. Exonic splicing enhancer possesses an activity to retain RNAs in the nucleus through a saturable nuclear retention factor.^40^ Four purine-rich exonic splicing enhancers in *TRA2β* exon 2 may be important for nuclear retention of *TRA2β4* to interact with Sp1 and other regulatory factors. In addition, RNA secondary structures, especially construction of stems, affected the RNA-protein-binding activity.^41^ Ultraconserved cDNA segments with AT-rich elements are resistant to secondary structure formation, keeping the segments open to allow regulatory factor binding.^42^ This would explain why Sp1 could bind preferentially to AT-rich exon 2 of *TRA2β4*. *TRA2β4* exon 2 contains predictive Sp1-binding sites (485-GGGG-488 and 515-ACGG-518). CentroidFold and M-FOLD programs indicate the presence of a stem-loop structure (449–488 nt) within exon 2, which includes one Sp1-binding site (485-GGGG-488). Mutations of this site (485-AAGG-488) disrupted the stem-loop structure and eliminated the association between Sp1 and *TRA2β4* exon 2. Thus, the consensus Sp1-binding site seems to be crucial for the interaction of *TRA2β4* exon 2 with Sp1.

Overexpression of full-length *TRA2β4* in HCT116 cells significantly reduced *CDKN1A* mRNA expression and accelerated their proliferation. The mutations (485-AAGG-488) completely eliminated these effects, again suggesting a crucial role of the consensus Sp1-binding site in the *TRA2β4*-mediated control of cell fate. Finally, we confirmed that colon cancer tissues expressed significantly higher amounts of *TRA2β4*.

The present study suggests that the *TRA2B* gene may control both apoptosis and senescence of colon cancer cells by generating different splice isoforms (*TRA2β1* and *TRA2β4*). *TRA2β4* RNA is likely to be a new anti-senescence factor working specifically in cancer cells. Cellular senescence is one of the important tumor-suppressive barriers.^30, 31, 32, 43^ It inhibits tumor cell proliferation and suppresses tumor cell motility, indicating that the induction of senescence results in the suppression of tumor cell growth, invasion and metastasis. Moreover, *TRA2β4* knockdown could facilitate cellular senescence even in p53-deficient cells. Thus, *TRA2β4* might be a novel tumorigenic ncRNA and a potential therapeutic target for colon cancer.

## Materials and methods

### Cell growth, apoptosis and senescence assays

Wild-type and p53^−/−^ HCT 116 cells were cultured in McCoy's 5A medium (Gibco, Grand Island, NY, USA) supplemented with 5% (vol/vol) heat-inactivated fetal bovine serum and antibiotics at 37 °C in 5% CO_2_. T84 was maintained in Dulbecco's modified Eagle's medium/F-12 1:1 mixture (Gibco) supplemented with 10% fetal bovine serum. HEK293T, HT29, RKO and CaCo-2 cells were cultured in Dulbecco's modified Eagle's medium with 10% fetal bovine serum. Human diploid fibroblasts (WI-38 and TIG-3) and SW480 cells were cultured in 10% fetal bovine serum-containing minimum essential medium. For the analysis of cell growth, appropriate numbers of HCT 116 or HEK293T cells were seeded in tissue culture plates, and the number of growing cells was counted using an automatic cell counter (Countess; Invitrogen, Carlsbad, CA, USA). Cell numbers on the plates were also assessed using the CellTiter96 AQueous Cell Proliferation Assay (MTS) (Promega, Madison, WI, USA). Apoptosis was evaluated by measuring cleaved caspase-9 and -3 levels by western blotting, and TUNEL analysis using the DeadEnd Colorimetric TUNEL system (Promega) according to the manufacturer's protocol. Percentages of TUNEL-positive cells in five different fields (400 nm^2^ each) were calculated. The data were obtained in three independent experiments. Cellular senescence was assessed by the expression of SA-β-gal using an SA-β-gal Staining Kit (Cell Signaling Technology, Danvers, MA, USA).

### Northern blot analysis

Locked nucleic acid-modified oligonucleotide probes targeting *TRA2β* transcripts (Gene Design, Osaka, Japan) were labeled with [^32^P]dCTP using recombinant terminal deoxynucleotidyl transferase (Life Technologies, Carlsbad, CA, USA). Oligonucleotide sequences using Northern blotting were listed in Supplementary Table S1. Samples of total RNA were separated in a 1% agarose gel containing 0.6 m formaldehyde and transferred to a nylon membrane filter. After prehybridization, the membrane was hybridized at 60 °C with the ^32^P-labeled probe overnight. After washing, bound probes were analyzed by BAS 1500 Image Analyzer (Fujifilm, Tokyo, Japan).

### Preparation of nuclear and cytosolic fractions

After HCT116 cells were incubated in cytosolic lysis buffer (10 mm Tris-HCl, pH 7.4; 100 mm NaCl; 2.5 mm MgCl_2_; 40 μg/ml digitonin) for 10 min, lysates were centrifuged at 2060 *g* for 8 min at 4 °C, and supernatants were collected as cytosolic extracts. The remaining pellets were washed two times with the cytosolic lysis buffer and lysed with RIPA buffer (10 mm Tris-HCl, pH 7.4; 150 mm NaCl; 1 mm EDTA; 1 mm dithiothreitol; 0.1% sodium dodecyl sulfate; 1% Nonidet P-40). After centrifugation at 21 000 *g* for 10 min at 4 °C, supernatants were collected as nuclear extracts. Cells lysed with RIPA buffer were used as whole-cell extracts.

### siRNAs

We used an siRNA (Hs_SFRS10_6 HP validated siRNA; Qiagen, Chatsworth, CA, USA) to knock down exon 3-containing *TRA2β1* and *TRA2β4* mRNAs. To silence selectively *TRA2β4*, exon 2-targeting siRNA was designed (Supplementary Table S1). A negative control siRNA (AllStars Negative Control siRNA) was obtained from Qiagen.

### Quantitative real-time reverse transcription–PCR

Total RNAs were extracted from cells using TRIzol reagent (Life Technologies). One microgram of isolated RNA was reverse-transcribed using a PrimeScript RT Reagent Kit (Takara, Otsu, Japan). *TRA2β1*, *TRA2β4* and *CDKNA1* mRNA levels were measured using SYBR Green Master Mix and Applied Biosystems 7500 Real-time System (Applied Biosystems, Foster City, CA, USA). The sequences of primer sets are provided in Supplementary Table S1. TissueScan Tissue qPCR Arrays (HCRT103) including cDNAs from paired normal and tumor tissues in 24 patients with adenocarcinomas of the colon were obtained from OriGene Technologies (Rockville, MD, USA), and *TRA2β4* levels in normal and tumor tissues were determined by qPCR. *TRA2β4* levels were measured by the comparative ΔΔCt method using *ACTB* mRNA as a control and expressed as values relative to the normal samples.

### Western blotting

Whole-cell lysates were prepared in a RIPA buffer (10 mm Tris-HCl, pH 7.4; 1% Nonidet P-40; 1 mm EDTA; 0.1% sodium dodecyl sulfate; 150 mm NaCl) containing a protease and phosphatase inhibitor cocktail (Roche Diagnostics Japan, Tokyo, Japan). Mouse monoclonal anti-α-tubulin (1:1000; Santa Cruz Biotechnology, Santa Cruz, CA, USA), anti-Tra2β (1:1000; Abcam, Cambridge, UK), anti-caspase-3 (1:1000; Cell Signaling Technology, Danvers, MA, USA), anti-cleaved caspase-3 (1:1000; Cell Signaling Technology), anti-PARP (1:1000; Cell Signaling Technology), anti-cleaved PARP (1:1000; Cell Signaling Technology), anti-p21 (1:1000; Santa Cruz Biotechnology), anti-p53 (1:1000; Santa Cruz Biotechnology), anti-β-actin (1:1000; Abcam) or anti-Sp1 (1:1000, Santa Cruz Biotechnology) antibody was used.

### Promoter activity assay

The 5′ flank of the human *CDKNA1* gene was cloned into the pGL3-basic luciferase reporter vector (Promega). In brief, the first PCR was performed using human genomic DNA as a template. The *CDKNA1* proximal promoter region was amplified using primer sets listed in Supplementary Table S1. The amplified products were subcloned into the pGL3-basic vector using *Hind*III and *Xho*I restriction sites. HCT116 cells (1.0 × 10^5^) were cultured on 24-well plates, and then pGL3 luciferase constructs with various site-directed mutation or deletion (100 ng) were co-transfected with pRL-CMV vector (100 ng) using Jet-PEI (Polyplus Transfection, Illkirch, France). Twenty-four hours after the transfection, cells were harvested and the firefly and *Renilla* luciferase activities were measured using the Dual-Luciferase Reporter Assay System (Promega).

### Chromatin immunoprecipitation assay

Chromatin immunoprecipitation assays were performed using the Chromatin Immunoprecipitation Assay Kit (Millipore, Billerica, MA, USA). Briefly, HCT116 cells were fixed with 1% formaldehyde in phosphate-buffered saline for 10 min and then washed two times with ice-cold phosphate-buffered saline. These cells were resuspended in sodium dodecyl sulfate lysis buffer, incubated for 10 min on ice and then sonicated. Immunoprecipitation was carried out overnight at 4 °C using 3 μg antibody against Sp1. Normal rabbit IgG was used to assess nonspecific reactions. Immune complexes were collected with protein A agarose/salmon sperm DNA. Crosslinking between proteins and DNA was reversed according to the manufacturer's protocol. Protein-bound DNA was extracted with phenol/chloroform/isoamyl alcohol. The extracted DNA was amplified by PCR (35 cycles; denaturing at 98 °C for 10 s, annealing at 55 °C for 30 s and extension at 72 °C for 1 min) using the following primers: for the *CDKNA1A* sequence between −560 and -226 bp, 5′-GGTGTCTAGGTGCTCCAGGT-3′ and 5′-GCACTCTCCAGGAGGACACA-3′ for the *CDKNA1-B* sequence between −325 and +51 bp, 5′-CAGCGCACCAACGCAGGCG-3′ and 5′-CAGCTCCGGCTCCACAAGGA-3′. The nuclear chromatin DNA from HCT116 cells (input) was used as a positive control for PCR.

### Biotin pull-down assay

PCR fragments containing the T7 RNA polymerase promoter sequence were used as templates for *in vitro* transcription. Biotinylated transcripts were prepared by using a MEGAscript T7 Kit (Life Technologies) and biotin-CTP (Perkin-Elmer Japan, Yokohama, Japan), and purified with ssDNA/RNA Clean and ConcentratorTM (Zymo Research, Orange, CA, USA). Biotin pull-down assays were carried out by incubating 40 μg of nuclear fractions with 1 μg of biotinylated transcripts in TENT buffer (10 mm Tris-HCl (pH 8.0), 1 mm EDTA, 250 mm NaCl and 0.5% Triton X-100) for 1 h at room temperature. Complexes were isolated with paramagnetic streptavidin-conjugated Dynabeads (Life Technologies), and bound proteins in the pull-down materials were analyzed by western blotting using an anti-Sp1 antibody.

### RIP assay

HCT116 cells were lysed with 25 mm Tris-HCl buffer (pH 7.5) containing 150 mm NaCl, 1 mm EDTA, 1% (v/v) Nonidet P-40, 5% (v/v) glycerol and 100 U/ml RNase inhibitor (Promega). Whole-cell extracts (500 μg protein) were incubated with protein A Sepharose beads precoated with 3 μg anti-Sp1 antibody (Santa Cruz Biotechnology) or control rabbit IgG for 1 h at 4 °C. After washing with NT2 buffer (50 mm Tris-HCl buffer, pH 7.4, containing 150 mm NaCl_2_, 1 mm MgCl_2_ and 0.05% Nonidet P-40), beads were incubated with 20 U of RNase-free DNase I (Life Technologies) in NT2 buffer for 15 min at 37 °C and further incubated in NT2 buffer containing 0.1% sodium dodecyl sulfate and 0.5 mg/ml proteinase K for 20 min at 55 °C. RNA in the IP materials was measured by qPCR.^2^

### Fluorescent *in situ* hybridization

Cells were fixed with 4% paraformaldehyde and permeabilized with 0.5% Triton X for 5 min. RNA probes were prepared using Fluorescein Isothiocyanate RNA Labeling Kit (Roche Diagnostic, Mannheim, Germany) according to the manufacturer's protocol. Then, cells were incubated for 16 h at 55 °C with 2 x SSC containing fluorescein isothiocyanate-labeled RNA probes. The cells were washed with 2 x SSC 55 °C for 30 min, the nuclei were stained using TO-PRO-3 (Life Technologies) and coverslipped with Vectashield (Life Technologies).
