# Supplementary material for: Ultraconserved region-containing Transformer 2β4 controls senescence of colon cancer cells
Source: Oncogenesis. 2016 Apr 4;5(4):e213–. doi: 10.1038/oncsis.2016.18 (PMC4848834; doi:10.1038/oncsis.2016.18)
Supplement: Supplementary Figures [file oncsis201618x1.pdf]

```
Mm    TGCATAT-CTAGTATTTTTTT----------AATCCAAAACTACGAAAAGGATTTTTTT
Rn    TGCATAT-CTAGTTTTTTTTTTTTTTTTTTTAAATCCAAAACTACAAAAAGGATTTTTTT
Hs    TGCATATTCTAGTTTCTTTTTTTTTTTTTTTAATCCAAATCAAC--AAAGGATTTTTTT
      ******* ***** * *****          ******** * **  **************

Mm    TT-CTAGTTTCAGTTTTGGCACTGGATTTTATCCTGGAGTTTTAAAATATTCTTCATCCT
Rn    T--CTAGTTTCAGTTTTGGCACTGGATTTTATCCTGGAGTTTTAAAATATTCTTCATCCT
Hs    TTTCTAGTTTCAGTTTTGGCACTGGATTTTATCCTGGAGTTTTAAAATATTCTTCATCCT
      *  *********************************************************

Mm    GTTCTTTTTCTATTAAGGTTAATGTTGAAGAAGGAAAATGCGGAAGTCGTCATTTGACAA
Rn    GTTCTTTTTCTATTAAGGTTAATGTTGAAGAAGGAAAATGCGGAAGTCGTCATTTGACAA
Hs    GTTCTTTTTCTATTAAGGTTAATGTTGAAGAAGGAAAATGCGGAAGTCGTCATTTGACAA
      ************************************************************

Mm    GTTTTATAAATGAGTATTTGAAGCTCAGGAATAAGTGAAGCTGAAATTTGAAAAAATAAA
Rn    GTTTTATAAATGAGTATTTGAAGCTCAGGAATAAGTGAAGCTGAAATTTGAAAAAATAAA
Hs    GTTTTATAAATGAGTATTTGAAGCTCAGGAATAAGTGAAGCTGAAATTTGAAAAAATAAA
      ************************************************************

Mm    AGAAAGAATGCATGCTAATTATCAGACCAGAAGTCCCACTTGTAGAATATTGAGCAATTT
Rn    AGAAAGAATGCATGCTAATTATCAGACCAGAAGTCCCACTTGTAGAATATTGAGCAATTT
Hs    AGAAAGAATGCATGCTAATTATCAGACCAGAAGTCCCACTTGTAGAATATTGAGCAATTT
      ************************************************************

Mm    GTGTGAAGTGGGGAAGATGAAAGAAGTCAGAATTTGAAACGGAAGAATGAAGAAAAGAAA
Rn    GTGTGAAGTGGGGAAGATGAAAGAAGTCAGAATTTGAAACGGAAGAATGAAGAAAAGAAA
Hs    GTGTGAAGTGGGGAAGATGAAAGAAGTCAGAATTTGAAACGGAAGAATGAAGAAAAGAAA
      ************************************************************

Mm    TAAAAATGAAGTTAAGATAAGAAGTAATCTGGAATCAGAAAGCACTACGCTAAGTAATTA
Rn    TAAAAATGAAGTTAAGATAAGAAGTAATCTGGAATCAGAAAGCACTACGCTAAGTAATTA
Hs    TAAAAATGAAGTTAAGATAAGAAGTAATCTGGAATCAGAAAGCACTACGCTAAGTAATTA
      ************************************************************

Mm    CTAGTCTGTTTATGTGTCCCTGTATATTTTGCTAAACATGCATGCATTATTTGTTTAATA
Rn    CTAGTCTGTTTATGTGTCCCTGTATATTTTGCTAAACATGCATGCATTATTTGTTTAATA
Hs    CTAGTCTGTTTATGTGTCCCTGTATATTTTGCTAAACATGCATGCATTATTTGTTTAATA
      ************************************************************

Mm    GAAGAA-GTATATACAGGGTAAAGAAGTGACTTCCAAGGGAATCGTTTAAA
Rn    GAAGAAAGTATATACAGGGTAAAGAAGTGACTTCCAAGGGAATCGTTTAAA
Hs    GAGGAAAATATATACAGGGTAAAGAAGTGCCTTCCAGGGGAAACGTTTAAA
      ** ***  ********************* ****** ***** ********
```

Fig. S1 Alignment of ultraconserved sequences (uc.138) in *TRA2B* genes.

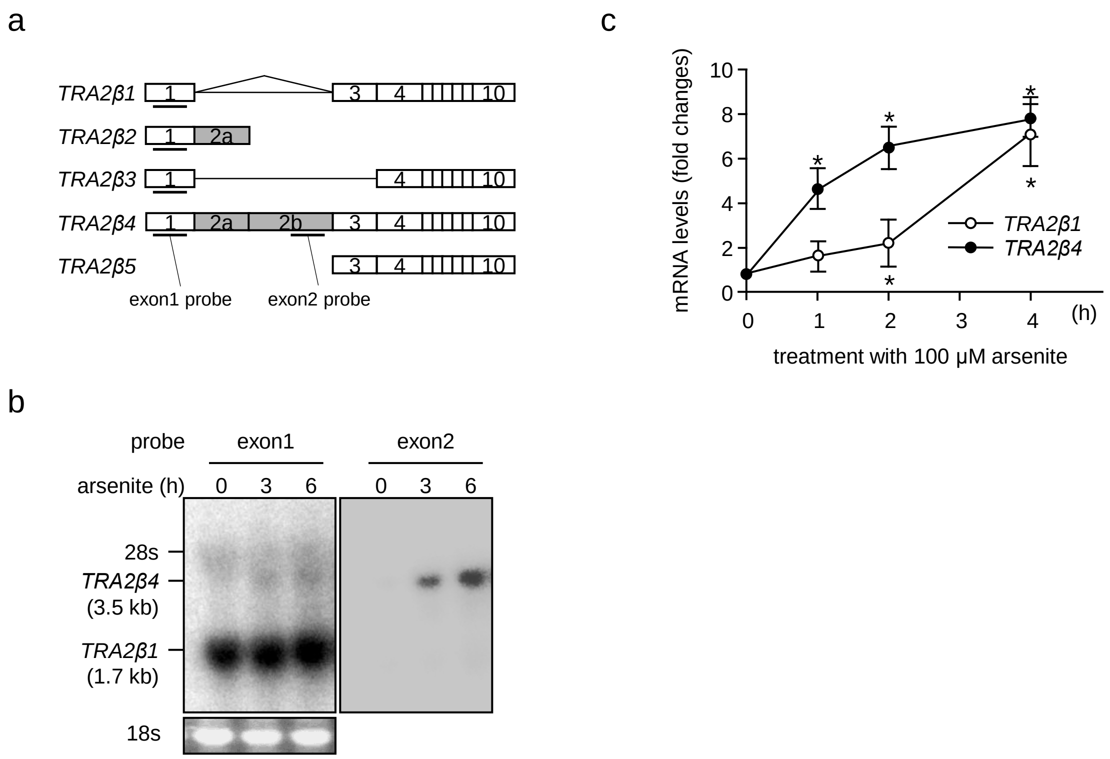

Fig. S2 Arsenite-stimulated expression of *TRA2β4* in HCT116 cells.

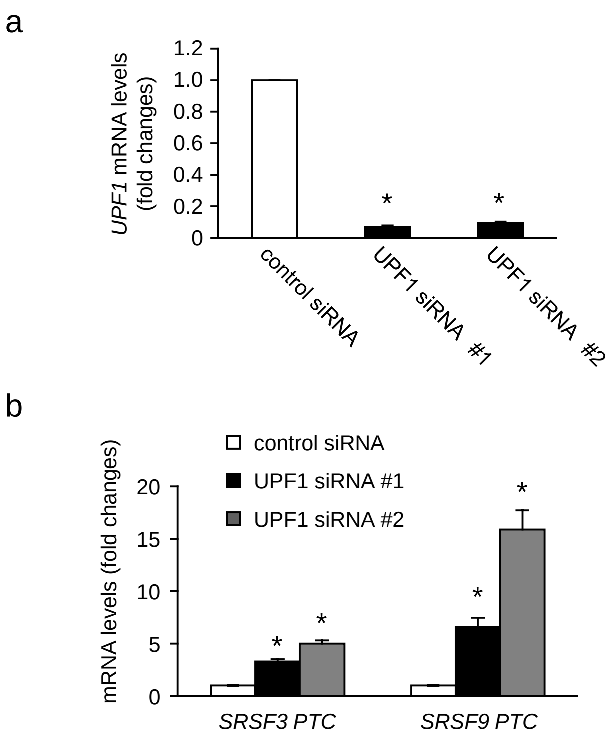

Fig. S3 UPF1 knockdown increases PTC variants of *SRSF3* and *SRSF9*.

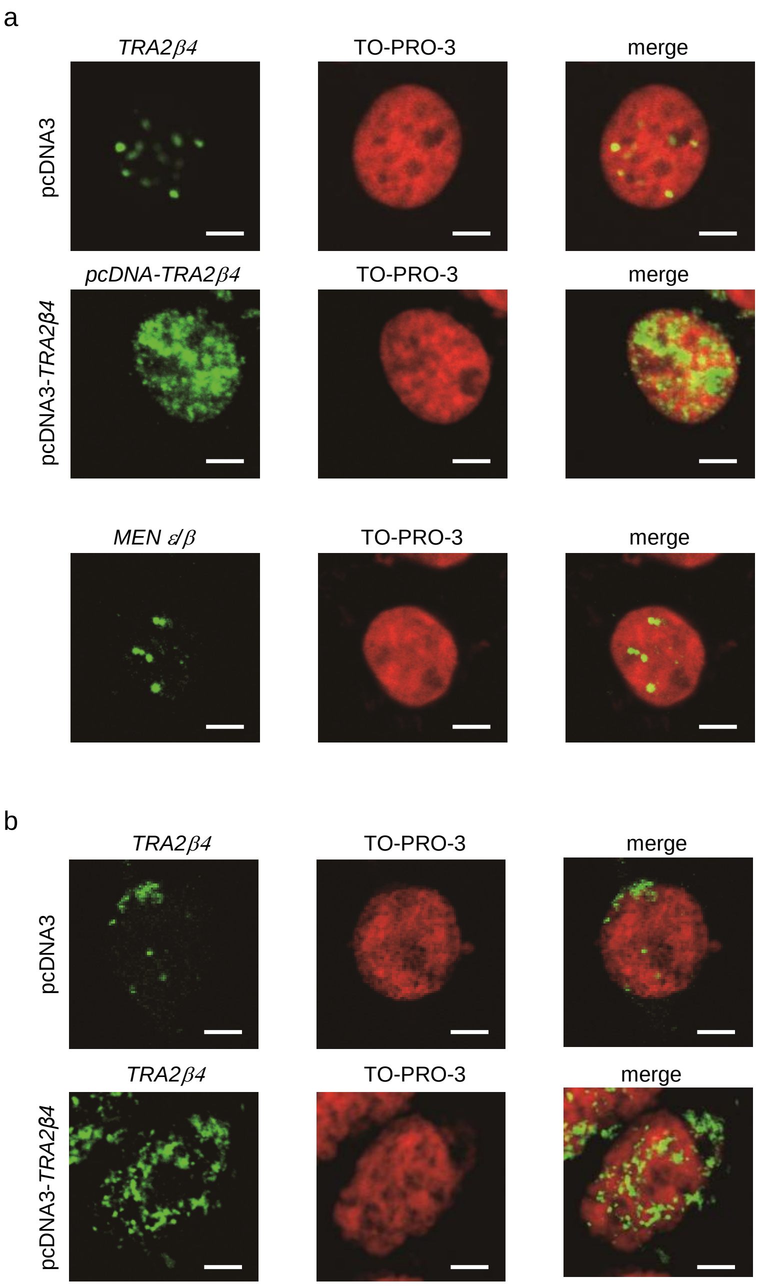

Fig. S4 Nuclear localization of *TRA2β4*.

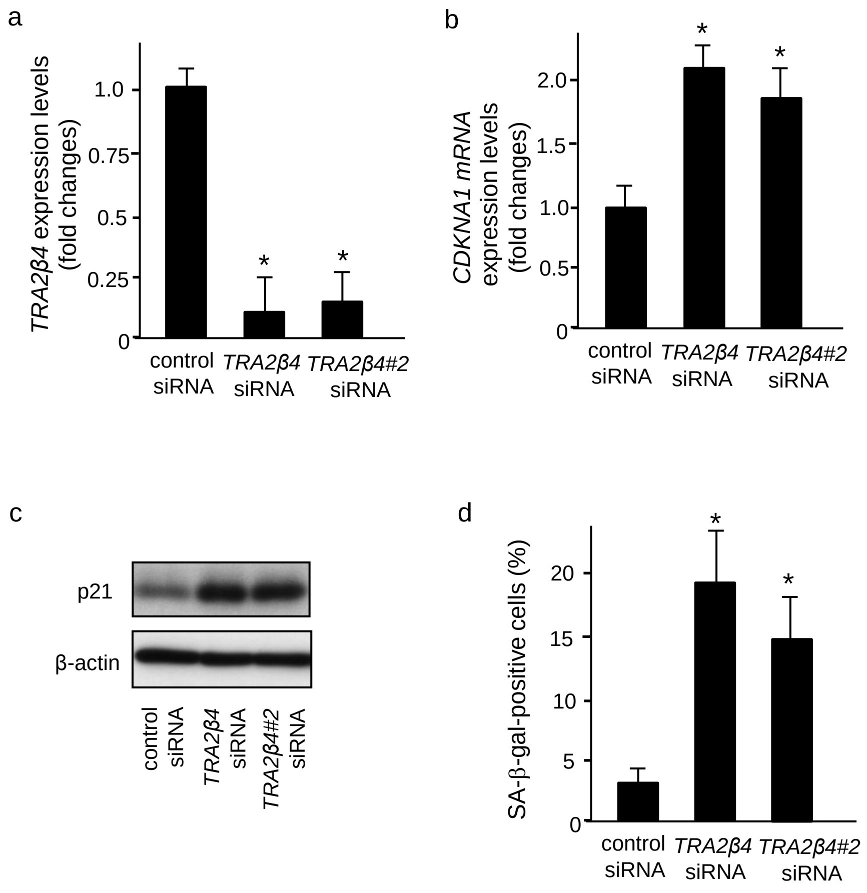

Fig. S5 *TRA2β4* knockdown increased expression of *CDKNA1* mRNA and p21 protein, and induced cellular senescence.

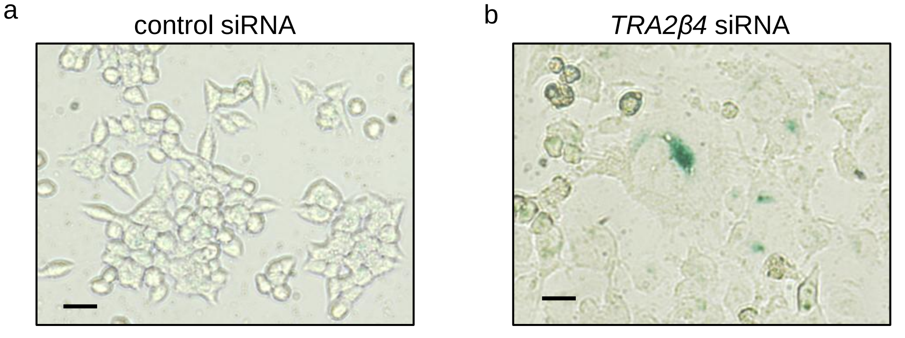

Fig. S6 *TRA2β4* knockdown facilitates cellular senescence in p53$^{-/-}$ HCT116 cells.

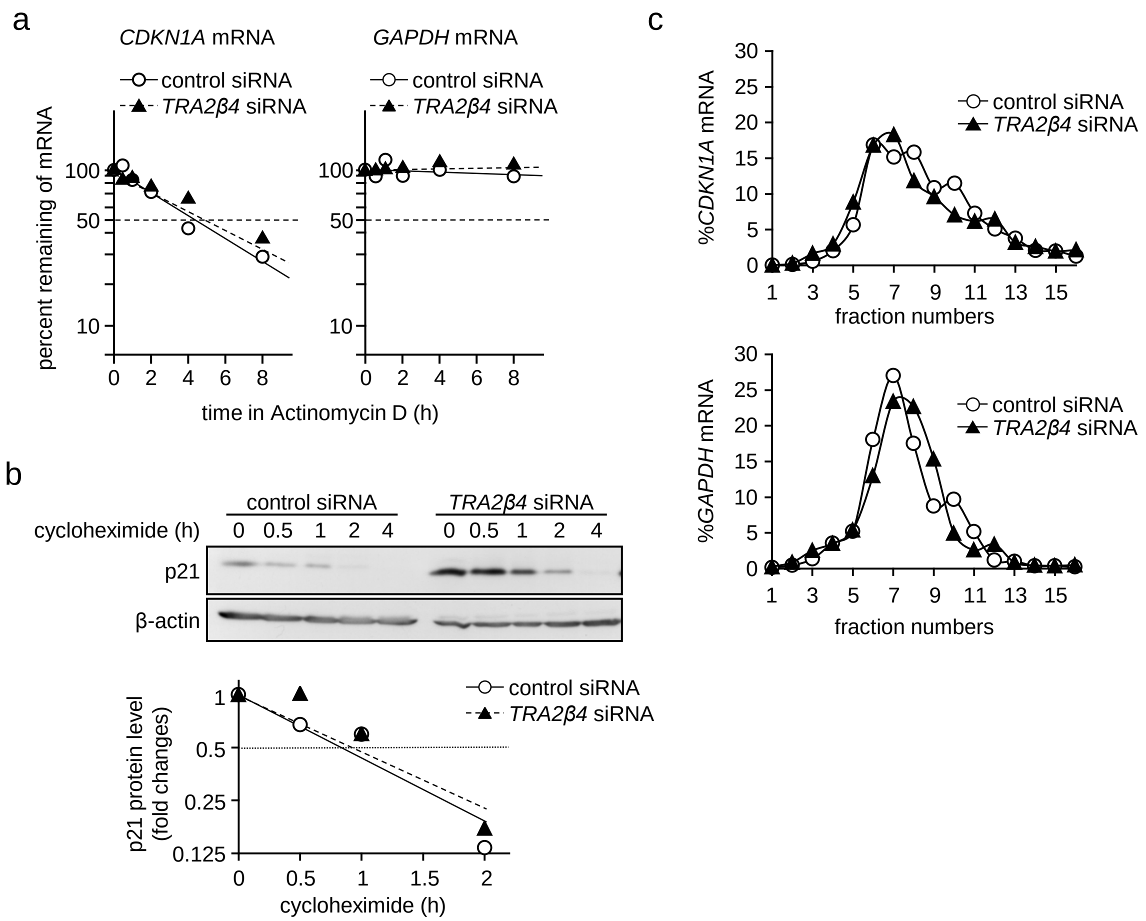

Fig. S7 *TRA2β4* knockdown does not affect stability of p21 protein or *CDKN1A* mRNA or translation rate of *CDKN1A* mRNA.

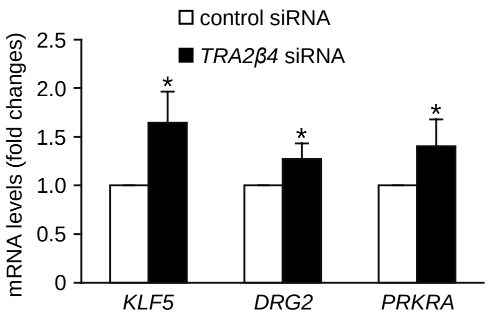

Fig. S8 *TRA2β4* knockdown up-regulates Sp1-mediated transcription of distinct genes.
