# Supplementary material for: Ultraconserved region-containing Transformer 2β4 controls senescence of colon cancer cells
Source: Oncogenesis. 2016 Apr 4;5(4):e213–. doi: 10.1038/oncsis.2016.18 (PMC4848834; doi:10.1038/oncsis.2016.18)
Supplement: Supplementary Information [file oncsis201618x2.doc]

**SUPPLEMENTARY FIGURE REGENDS**

**Fig. S1** **Alignment of ultraconserved sequences (uc.138) in *TRA2B* genes.** Ultraconserved segments (461 bp) in human (Hs), mouse (Mm), and rat (Rn) *TRA2B* genes are shown by a square. This segment includes all of exon 2 as shown in gray (276 bp) and neighboring introns. The consensus bases are indicated by asterisks (*).

**Fig. S2 Arsenite-stimulated expression of *TRA2β4* in HCT116 cells.** (**a**) Diagram of five splice variants generated from the *TRA2B* gene. Usage of each exon is indicated by Arabic numbers. Filled boxes denote the ultraconserved region. Probes used for Northern hybridization are represented by horizontal bars. (**b**) Total RNAs were extracted from HCT116 cells before (0 h) or at the indicated h after treatment with 100 μM sodium arsenite. Northern hybridization with the oligoDNA probe targeting exon 1 showed that HCT116 cells constitutively expressed 1.7-kb *TRA2β1* mRNA and induced expression of 3.5-kb *TRA2β4* in response to arsenite, as previously shown in AGS cells.^13^ Blots were stripped by boiling and reprobed with the probe targeting exon 2. This probe more clearly showed that arsenite stimulated expression of 3.5-kb *TRA2β4* within 3 h. Signals corresponding to the other isoforms could not be detected by Northern hybridization. As a loading control, *18S* rRNA visualized by ethidium bromide staining was used. (**c**) Time-dependent changes in *TRA2β1* and *TRA2β4* levels after treatment with 100 μM sodium arsenite were examined by qPCR using specific primer sets. The data are shown as fold changes compared with those in untreated control cells (0 h). Values are means ±SD from 3 independent experiments. ^*^Significantly increased by ANOVA and Bonferroni test (*P* < 0.05) compared with untreated control cells.

**Fig. S3 UPF1 knockdown increases PTC variants of *SRSF3* and *SRSF9*.**

(**a**) Treatment of HCT116 cells for 48 h with two different UPF1 siRNAs (#1 and #2) reduced *UPF1* mRNA levels < 10% of those in control siRNA-treated cells. (**b**) Levels of *SRSF3* and *SRSF9* PTC variants in the siRNA-treated cells were measured by qPCR using *GAPDH* as an endogenous quantity control. Data were expressed as fold-changes relative to the levels in control siRNA-treated cells. Values are means ± SD from 3 independent experiments. ^*^Significantly increased by ANOVA and Bonferroni test (*P* < 0.05) compared with control siRNA-treated cells.

**Fig. S4** **Nuclear localization of *TRA2β4*.** HeLa (**a**) and HEK293T (**b**) cells were transiently transfected with a construct encoding human full-length *TRA2β4* cDNA or pcDNA3.1 vector alone. RNA-FISH using a specific *TRA2β4* probe revealed that both endogenous and transfected *TRA2β4* were localized predominantly in nuclei (green) similar to a nuclear-retained non-coding RNA (*MEN ɛ/β*) (Sunwoo, H. et al *Genome Res* 19, 347-59, 2009). DNA was counterstained with TO-PRO-3 (red). Bars represent 5 μm.

**Fig. S5 *TRA2β4* knockdown increases *CDKNA1* mRNA and p21 expression and cellular senescence.** (**a**) Treatment of HCT116 cells for 48 h with two different *TRA2β4* siRNAs (*TRA2β4* and *TRA2β4#2*) reduced *TRA2β4* levels < 20% of those in control siRNA-treated cells. (**b**) Levels of *CDKNA1* mRNA in the siRNA-treated cells were measured by qPCR using *GAPDH* as an endogenous quantity control. Data were expressed as fold-changes relative to the levels in control siRNA-treated cells. (**c**) After the transfection for 48 h, amounts of p21 were measured by Western blotting. (**d**) The cells were stained with SA-β-gal and then one hundred cells per individual sample in 3 independent fields were measured. Values are means ± SD from 3 independent experiments. ^*^Significantly increased by ANOVA and Bonferroni test (*P* < 0.05) compared with control siRNA-treated cells.

**Fig. S6** ***TRA2β4* knockdown facilitates cellular senescence in p53^-/-^ HCT116 cells.** After p53^-/-^ HCT116 cells were transfected with 10 nM control (**a**) or *TRA2β4* siRNA (**b**) for 72 h, they were assayed for SA-β-gal staining. Scale bars, 10 μm

**Fig. S7** ***TRA2β4* knockdown does not affect stability of p21 protein or *CDKN1A* mRNA or translation rate of *CDKN1A* mRNA.** (**a**) HCT116 cells were treated with 10 nM control or *TRA2β4* siRNA for 48 h. They were then incubated with 2 μg/ml actinomycin D for the indicated times. The amounts of *TRA2β4* and *GAPDH* mRNAs were measured by qPCR using *18S* rRNA for normalization. The amounts remaining are expressed as percentages of those in untreated control cells (0 h) and plotted on a semi-logarithmic scale. (**b**) HCT 116 cells transiently transfected with control or *TRA2β4* siRNA were treated with 25 μg/ml of cycloheximide for the indicated times. Cell lysates were subjected to Western blots using an anti-p21 antibody (*upper panel*). Image J software (NIH, Bethesda, MD) was used for quantification of Western blots (*lower panel*). (**C**) Forty-eight hours after transfection with either control siRNA (○) or *TRA2β4* siRNA (▲), HCT116 cells were incubated with 100 μg/ml of cycloheximide for 5 min. Cytoplasmic lysates prepared from these cells were centrifuged through 10–50% linear sucrose gradients and collected in 16 fractions. Levels of *CDNK1A* and *GAPDH* mRNAs in each fraction were measured by qPCR (Abdelmohsen *et al*, *Proc Natl Acad Sci U S A* 105(2):20297-302, 2008) and plotted as a percentage of the total *CDKN1A* or *GAPDH* mRNA levels. *TRA2β4* knockdown did not change the stability of p21 or *CDKN1A* mRNA or the polysome pattern of *CDKN1A* mRNA.

**Fig. S8** ***TRA2β4* knockdown up-regulates Sp1-mediated transcription of distinct genes.** After treatment with control or *TRA2β4* siRNA for 48 h, total RNAs were extracted and then subjected to qPCR measurement of *Kruppel-like factor 5* (*KLF5*), *developmentally regulated GTP-binding protein 2* (*DRG2*), and *protein kinase, interferon-inducible double stranded RNA dependent activator* (*PRKRA*) mRNA levels. Sp1 is crucial for transcription of these.^24-26^ Significant up-regulation of *KLF5*, *DRG2*, and *PRKRA* mRNA expression after *TRA2β4* knockdown suggests that *TRA2β4* may facilitate Sp1 occupancy of its binding elements in these gene promoters and up-regulate their expression. Data are expressed as fold-changes relative to the levels in control siRNA-treated cells. Values are means ± SD from 4 independent experiments. ^*^Significantly increased by ANOVA and Bonferroni test (*P* < 0.05) compared with control siRNA-treated cells.

**Supplementary Table S1. Primer sets used for reverse transcription (RT)-PCR and qPCR, oliginucleotide sequences for siRNA, Northern blotting and RNA-FISH**

|  |  |  |  |
| --- | --- | --- | --- |
| Primers |  |  |  |
| Targets |  | Primer Sequences (5' - 3') |  |
| *TRA2β4* | forward | AGGAAAATGCGGAAGTCGTC |  |
|  | reverse | CTGGAAGCAGAACGGGATTCTTA |  |
| *TRA2β1* | forward | CGGCGAGCGGGAATCCCG |  |
|  | reverse | GACATGGGAGAATGGCTGTGGC |  |
| *GAPDH* | forward | AGCCACATCGCTCAGACAC |  |
|  | reverse | GCCCAATACGACCAAATCC |  |
| *SFRS3 ref* | forward | GTGAAAAAAGAAGTAGAAATCGTGG |  |
|  | reverse | CTCCTTCTTGGAGATCTGCGACGAG |  |
| *SFRS3 PTC* | forward | TCCACCTCGTCGCAGAGTCACCATC |  |
|  | reverse | TCATGTGAAACGACACCAGCCAAGC |  |
| *SFRS9 ref* | forward | GAGTTCTTGTTTCAGGACTTCCTCC |  |
|  | reverse | CATGAGAGCGGAATTTGGTGTCATC |  |
| *SFRS9 PTC* | forward | CTTTCATGACATCAGGACTTCCTCC |  |
|  | reverse | CATGAGAGCGGAATTTGGTGTCATC |  |
| *UPF1* | forward | GCTGAAGGAGTCCCAGACTCAA |  |
|  | reverse | CCCTTTGTACCGCAGGCATATC |  |
| *CDKN1A* | forward | GCAGGGGACAGCAGAGGAAG |  |
|  | reverse | AGAAGATCAGCCGGCGTTTG |  |
| *UBC* | forward | CCACTCTGCACTTGGTCCTG |  |
|  | reverse | TGCAACAACTTTATTGAAAGGAAA |  |
| *U6* | forward | CTCGCTTCGGCAGCACA |  |
|  | reverse | AACGCTTCACGAATTTGCGT |  |
| *18S* | forward | CCCTATCAACTTTCGATGGTAGTCG |  |
|  | reverse | CCAATGGATCCTCGTTAAAGGATTT |  |
| *ACTB* | forward | ATTGCCGACAGGATGCAGA |  |
|  | reverse | GAGTACTTGCGCTCAGGAGGA |  |
| *pre-GAPDH* | reverse | CCCATACGACTGCAAAGACC |  |
|  |  |  |  |
| (for *in vitro* transcription ) | | |  |
| *TRA2β4* | forward | ATGAGCGACAGCGGCGAGCAG |  |
|  | reverse | [T7]GACCGTGACCGGGTATAATG |  |
| *TRA2β1* | forward | ATGAGCGACAGCGGCGAGCAG |  |
|  | reverse | [T7]GAACGCCTAGACTGCTGGTC |  |
| *MEN ε/β* | forward | TAGTTGTGGGGGAGGAAGTG |  |
|  | reverse | [T7]TGGCATGGACAAGTTGAAGA |  |
| *All reverse primers include [T7: ccaagcttctaatacgactcactatagggaga] promoter sequence | | | |
|  |  |  |  |
| (for *CDKN1A* promoter cloning ) | | |  |
| CDKN1A (-2688) | forward | AAAAACTCGAGGGCTGCCTCTGCTCAATAATGTTCT |  |
| CDKN1A (-774) | forward | AAAAACTCGAGGAGTGTAGGGTGTAGGGAGATTGGT |  |
| CDKN1A (-163) | forward | AAAAACTCGAGGCTGGAACTCGGCCAGGCTCAGCTG |  |
| CDKN1A (+31) | reverse | AAAAA AAGCTTACTGACTTCGGCAGCTGCTCACACC |  |
|  |  |  |  |
| (for chromatin immunoprecipitation ) | | |  |
| CDKN1A primer 1 | forward | GGTGTCTAGGTGCTCCAGGT |  |
|  | reverse | GCACTCTCCAGGAGGACACA |  |
| CDKN1A primer 2 | forward | CAGCGCACCAACGCAGGCG |  |
|  | reverse | CAGCTCCGGCTCCACAAGGA |  |
|  |  |  |  |
| Sequence of siRNAs | |  |  |
| Name |  | Sequence (5'-3') | Target |
| *TRA2β4* siRNA  *TRA2β4* siRNA#2 |  | CACUUGUAGAAUAUUGAGCAA  UUGTAGAAUAUUGAGCAAUUU | exon 2  exon2 |
| UPF1 siRNA #1 |  | GAGACAGUCCUGGAGUGCUACAACU | exon 4 |
| UPF1 siRNA #2 |  | GACAGUUACCUUGGUGACGAGUUUA | exon 23 |
| Sp1 siRNA#1  Sp1 siRNA#2 |  | Santa Cruz Biotech. (h)  Santa Cruz Biotech. (h2) | -  - |
| Oligonucleotide sequences using RNA-FISH and Northern blotting | | |  |
| Name |  | Sequence (5'-3') | Target |
| LNA probe targetting *TRA2β4** |  | ttGctcAataTtctAcaaGtg | exon 2 |
| LNA probe targetting *TRA2β1** |  | cagAacgGgatTcccGctcGccgTag | exon 1, 3 |
| TRA2β ex1 |  | GCCGCTGTCGCTCATGACTCCTGGC  TGCTGTCGCCGGTCGATGTGCTTCA  ATCGAAGCTGCCAACCTCTTGCACCTTCCTTAAGG | exon 1 |
| *Capital letters mean locked-nucleic acids | | |  |
